# Malaria in Afghanistan: Challenges, efforts and recommendations

**DOI:** 10.1016/j.amsu.2022.104424

**Published:** 2022-08-17

**Authors:** Javeria Arif Siddiqui, Humna Aamar, Amna Siddiqui, Mohammad Yasir Essar, Muhammad Abdullah Khalid, Sayed Hamid Mousavi

**Affiliations:** Faculty of Medicine, Sindh Medical College, Jinnah Sindh Medical University, Karachi, Pakistan; Department of Medicine, Karachi Medical and Dental College, Karachi, 74700, Pakistan; Kabul University of Medical Sciences, Kabul, Afghanistan; Afghanistan National Charity Organization for Special Diseases, Kabul, Afghanistan; Faculty of Medicine, Dow Medical College, Dow University of Health Sciences, Pakistan; Medical Research Center, Kateb University, Kabul, Afghanistan; Afghanistan National Charity Organization for Special Diseases, Kabul, Afghanistan

## Abstract

Malaria, a vector borne disease that can quickly become life-threatening, has become endemic to many countries, in particular Afghanistan. Ranking third for world's highest burden of malaria, Afghanistan has found itself in a downward spiral, burdened by outbreaks of not only malaria, but dengue, watery diarrhea and measles as well. The civil conflict and lack of healthcare services present compounded with the COVID-19 pandemic resulted in a five-to tenfold increase of malarial incidence in the past years. Increased refugee shuttling and fluctuating environmental conditions have allowed proliferation of malarial vectors, with restricted access to treatment impeding elimination of malaria as well. Although efforts like larvicides, indoor spraying and initiatives like the Sehatmandi project have been made to control the spread of malaria, further efforts focusing on more sustainable and economical preventative measures are essential. Thus, efforts on both individual and global levels, more research and maintenance of control measures, are necessary to eliminate outbreaks and risks of resurgence.

## 1 Introduction

Malaria is an acute life-threatening disease, spread through the bite of infected female Anopheles mosquito, which transmits the parasitic protozoon, Plasmodium, into the human body [1]. Four species of Plasmodium infect humans: P. falciparum, P. vivax, P. ovale, and P. malariae [2]. In Afghanistan, P. falciparum and P. vivax are the major causes of malarial incidence, the latter being less common and less severe [3]. Symptoms of malaria arise after 10–15 days and consist of cyclic fevers, headaches, gastrointestinal and respiratory distress, anaemia, body pain, and in the worst cases, coma or death [1,4]. Timely treatment with anti-malarial drugs can resolve these symptoms, and prevention methods like insecticide spraying or insecticide sprayed nets can be used to reduce transmission [1].

With a deteriorating healthcare system, mass relocations and a civil crisis afoot, malaria has become endemic in Afghanistan, with over 150,000 cases being reported in 2019 [5]. Accounting for 11% of cases in the Eastern Mediterranean region, Afghanistan places third for the world's highest disease burden of malaria [3]. The civil conflict and lack of healthcare services present, compounded with the COVID-19 pandemic resulted in a five-to tenfold increase of malarial incidence in the past years. Many symptoms of malaria overlap with COVID-19 symptoms, such as fever, body pain, respiratory distress, etc. [4] Consequently, during the period of the pandemic, misdiagnosis due to inadequate diagnostic tools (i.e., laboratory testing) and nonspecific symptoms may have occurred and resulted in subsequent mistreatment and further transmission of both diseases [4].

Transmission of malaria occurs seasonally from June to November, and regional incidence varies based on human population density, behaviours, ecological, climate, agriculture, and access to health services for detection and treatment of malaria [5]. Higher prevalence occurs in low-altitude regions where rice is cultivated and cattle farming occurs, due to better environment for and zoophilic nature of the disease vectors [6]. A majority of the total malaria cases in the country occur in 6 provinces bordering Pakistan; Nangarhar, Laghman, Kunar, Nooristan, Khost, and Paktika, with the Nangarhar province encompassing more than 45% of cases in 2019 [5]. A recent outbreak of malaria reported from the Alishang district of Laghman province, a key endemic area, was supplied with anti-malarial medication provided by the World Health Organisation (WHO) [7]. Although 8 health facilities in this area received this aid, further intervention is integral to address the gaps in a system burdened by outbreaks of not only malaria, but dengue, watery diarrhea and measles as well [7].

Therefore, since malaria is a curable and preventable disease, the question remains, why is malarial incidence so high in Afghanistan? This commentary aims to answer this question by giving an overview of the current situation of malarial incidence in Afghanistan, the challenges faced by the government and people in eradicating this disease, and suggestions to alleviate these problems.

## 2 Challenges

Amongst the backdrop of political instability and conflict, Afghanistan shares the world's third-highest malaria burden after African countries [8]. A lack of malaria prevention measures, such as vector control and public awareness, has resulted in re-emergence of malaria outbreaks. With 60% of the population living in endemic areas, and nearly half a million malaria cases per year, the burden of malaria in Afghanistan demands immediate attention [9]. In Afghanistan, 95% of malaria cases are attributed to Plasmodium falciparum (P.f.) and 5% to Plasmodium vivax (P.v.) [10]. The state is vulnerable to an increased prevalence of Malaria cases because of amplified refugee shuttling than before due to the recent stroke of political instability in the region [3]. Fluctuating environmental conditions have provided a favourable breeding ground to the malarial vectors [5]. Flourishing in river pools, river edges, and rice fields, the sporogonic cycle of malarial vectors is temperature-dependent, resulting in fluctuating transmission rates of certain species and a constant threat of infection [9]. Furthermore, lack of availability of epidemiological data challenges the setup of an improved surveillance system and hampers efforts to reduce incidence of disease. Inadequate implementation of measures to control its spread has markedly accelerated malaria transmission in the country. Moreover, restricted access to treatment and preventative measures is also one of the obstacles ensuing a major proportion of the population residing in rural and remote areas. Whilst the most effective prevention method for malaria includes vector control via insecticide treated nets (ITNs), a study in 2015 revealed that only 26% of people owned at least one ITN and only 3% owned enough to cover all household members [3,8]. Notably, limited financial support to cover up all the components of the National strategy, particularly inefficient support, and incompetent staff at service delivery and programme management levels act as a significant barrier to the implementation of P.f. eradication programmes in provinces more prone to malaria outbreaks. Also, fragile monitoring systems especially the quality assurance of laboratory services concomitant with poor health-seeking behaviours in communities of the country temporally exacerbate the spread of malaria [3].

## 3 Efforts and recommendations

To control the spread of malaria, insecticide-treated nets (ITNs), larvicides, indoor spraying, and other biological and environmental methods have been used [11]. Efforts to lower this burden began in the 1950s, through a vertical program implemented by the government [6]. This plan included residual indoor spraying of insecticides and antilarval measures. Due to conflict in Afghanistan, only a few measures of the original plan were implemented by the 1990s [6]. Afterwards, basic health services like malaria diagnosis and treatment were carried out by non-governmental organisations (NGOs) and UN agencies [6]. Use of prevention methods like insecticide-treated bed nets (ITNs), treated sheets and curtains, mosquito repellants, and pyrethrum coils became more widespread [6,11]. These methods reduced blood feeding and entry of mosquitoes into the home, thereby reducing malaria incidence [6].

Current efforts to lower the burden focus on better quality of diagnosis and treatment, implementing sustainable and economical preventive measures, and a strengthened surveillance system, requiring community participation [11]. In 2013, the Community-Based Malaria Management (CBMM) strategy was introduced, which focuses on rapid diagnostic testing (RDT) and delivering more timely treatment to all communities nationwide [5]. Since 2016, more than 30,000 community health workers (CHWs) were trained on malaria case management, RDT use and distribution of ITNs [5]. In 2015, the Global Fund and UNDP began a six-year initiative, known as the Sehatmandi project [12]. This project focuses on providing HIV, TB, and malaria services in Afghanistan [12]. While providing funding for the CBMM strategy, this project has also begun early diagnostic malaria testing in rural areas through the delivery of 300,000 rapid malaria testing kits and training 26,000 CHWs in testing and treating malaria [13]. The Sehatmandi project and WHO collectively distributed 4.1 million ITNs to malaria prone communities [3,13]. WHO has also provided technical support to improve surveillance and provide information materials to increase public awareness about malaria [3]. Recently in 2021, the Global Fund provided funding to support the COVID-19 pandemic response and mitigate the impact of the pandemic on HIV, TB and malaria programmes [12].

Despite these efforts, malaria incidence remains high in Afghanistan. This is due to insecurity in many areas, limited skilled staff, limited financial support, and weakness in the monitoring system [3]. Thus, integration of malaria diagnosis and treatment into routine healthcare delivery is essential [11]. National strategies should broaden to include the health, agricultural, and private sectors to better promote malaria control through diagnosis and treatment, address vector control strategies and prevention [11]. While many ITNs have been distributed, only 3% of households have enough to cover all the members [3]. Standardized systems are required to monitor malaria drug efficacy and insecticide resistance, and address the shortage of ITNs [3,11]. Research would help identify more vulnerable populations, thus increasing delivery of testing and treatment to them [11]. Though, currently, such systems would require a greater capacity of management staff [3]. To reduce incidence of malaria, vector control through management of potential breeding sites (i.e. rice fields), and increasing access to anti-malaria interventions like ITNs, indoor spraying, diagnostics and effective drugs is integral [5,13]. Such control measures need to be maintained for 15–20 years or until methods of eradication are developed to eliminate the risk of resurgence [13].

## 4 Conclusion

Despite current efforts to control and reduce the burden of malaria in Afghanistan, incidence rates remain immensely high. It is therefore imperative to allocate global support to alleviate the disease burden of a country overwhelmed by political instability, multiple disease outbreaks, and an immensely fragile healthcare system. Without more financial support and improved control and surveillance measures, large populations in Afghanistan remain at high risk for disease. Thus, immediate steps must be taken on both individual and global levels to ameliorate the burden of malaria in Afghanistan.

## Ethical approval

N/A.

## Sources of funding

None.

## Author contribution

Mohammad Yasir Essar conceived the concept of the paper. Javeria Arif, Humna Aamar, Amna Siddiqui, and Abdullah Khalid wrote the first draft of the manuscript. Mohammad Yasir Essar, and Sayed Hamid Mousavi revised the second draft and made critical comments. All authors read and approved the final draft.

## Conflicts of interest

None declared.

## Consent

N/A.

## Registration of research studies

1. Name of the registry: N/A
2. Unique Identifying number or registration ID: N/A
3. Hyperlink to your specific registration (must be publicly accessible and will be checked): N/A

## Guarantor

N/A.

## Provenance and peer review

Not commissioned, externally peer reviewed.

## Declaration of competing interest

None.
